# Beyond Appearances: The Evolving Landscape of Functional Mitral Regurgitation

**DOI:** 10.1016/j.cjco.2025.10.012

**Published:** 2025-10-29

**Authors:** Xinyi Huang, Binni Cai, Simei Chen, Shufen Huang, Xu Chen, Maolong Su, Yan Wang

**Affiliations:** a Department of Echocardiography, Xiamen Cardiovascular Hospital of Xiamen University, School of Medicine, Xiamen University, Xiamen, China; b Department of Cardiology, Xiamen Cardiovascular Hospital of Xiamen University, School of Medicine, Xiamen University, Xiamen, China; c Department of Cardiac Function, Xiamen Cardiovascular Hospital of Xiamen University, School of Medicine, Xiamen University, Xiamen, China

**Keywords:** ventricular functional mitral regurgitation, left bundle branch pacing, atrial functional mitral regurgitation

**Functional mitral regurgitation (FMR) is a complex entity requiring tailored diagnostic and therapeutic approaches. This case uniquely illustrates how distinct FMR mechanisms can manifest sequentially in a single patient.**

## Clinical Case

A 77-year-old woman presented to an external hospital emergency department in 2018, following a sudden syncopal episode. Initial evaluation revealed bradycardia (heart rate 36-40 beats per minute) with a junctional escape rhythm. She was subsequently transferred to Xiamen Cardiovascular Hospital of Xiamen University. An admission electrocardiogram (ECG) demonstrated multisource ventricular tachycardia and ventricular escape rhythm, prompting urgent transvenous pacemaker placement via the right femoral vein to the right ventricle.

Holter monitoring (during intrinsic sinus rhythm and pacing) revealed first-degree atrioventricular (AV) block and complete left bundle branch block (CLBBB; QRS duration, 163 ms). Transthoracic echocardiography (TTE) showed left ventricular (LV) enlargement (end-diastolic diameter, 54 mm; LV end diastolic diameter indexed to body surface area, 39.13 mm/m^2^), severe LV systolic dysfunction (ejection fraction [EF], 25%), septal flash, apical rocking, and moderate mitral regurgitation (MR). Laboratory findings confirmed heart failure with an elevated N-terminal pro-B-type natriuretic peptide (NT-proBNP) level of 15,788 pg/mL.

**Figure 1:**
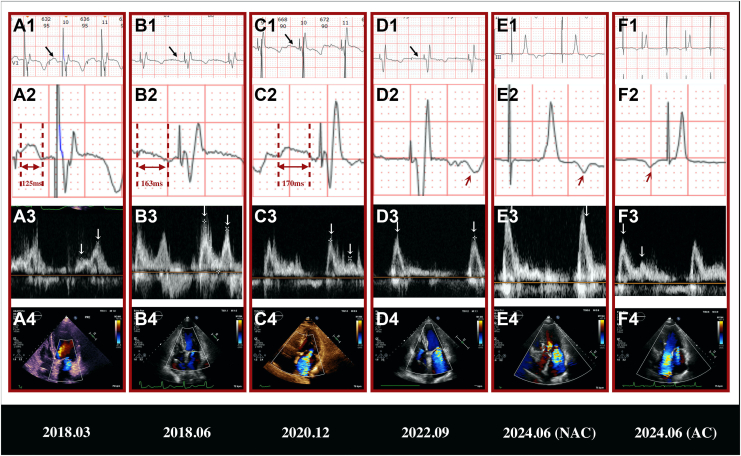
Serial follow-up electrocardiogram (ECG) and transthoracic echocardiography parameters. (**A1-D1, black arrows**) ECG V1 leads showed progressive P-wave flattening culminating in (**D1**) unrecognizable P waves in 2022. In 2024, ECG III leads showed unrecognizable P waves at lower output (5V/0.4 ms; paced AV delay, 150 ms), revealing no atrial capture (AC). (**F1**) At higher output (7V/1.0 ms, paced AV delay, 350 ms), anterograde P waves were restored with AC. (**A2, B2, C2**) Doubled-amplitude (20 mm/mV) demonstrated prolonged P-wave durations and (**D2, E2, red arrow**) the appearance of abnormal retrograde P' waves in 2022 and 2024 (no AC), (**F2, red arrow**) anterograde P waves in 2024 (AC). (**A3-F3**) Corresponding transmitral inflow Doppler patterns (E/A waves). (**A4-D4**) Progressive worsening of mitral regurgitation (MR) severity on follow-up TTE. (E4) Moderate-to-severe systolic MR combined with diastolic MR without AC compared to (**F4**) moderate systolic MR with AC.

One week later, after excluding myocardial ischemia and other secondary causes via coronary angiography and additional testing, the patient underwent dual-chamber permanent pacemaker implantation. A Medtronic 3830 lead (Minneapolis, MN) was positioned in the basal right ventricular septum for left bundle branch pacing, with atrial leads implanted in the right atrial appendage. Postoperative ECG demonstrated a narrow paced QRS complex (QRS duration, 91 ms). Repeat TTE 2 days post-implantation revealed reduced MR severity, improved LV systolic function (EF, 44%), and enhanced cardiac mechanical synchrony (systolic dyssynchrony index) decreased from 7.29% to 1.98%). Follow-up TTE at 3 months showed only mild MR (Fig. 1B4), normalized LV dimensions, and further recovery of LV systolic function (EF, 64%). The patient showed no limitations in ordinary physical activity, and improvement of New York Heart Association (NYHA) functional class, from III to I.

Subsequent annual post-discharge surveillance revealed progressive worsening of MR despite stable LV size and systolic function. The patient began to experience mild exertional dyspnea. NYHA function was rated as class II in 2022. TTE demonstrated left atrial (LA) enlargement and evolving transmitral inflow patterns: distinct E and A waves in 2020 (with A-wave truncation due to a short PR interval; Fig. 1C, panel 3) progressing to fusion by 2022 (Fig. 1D3). Concurrent ECG changes included flattened P waves in V1, prolonged P-wave duration, shortened PR segment, and ultimately, the emergence of abnormal retrograde P' waves following the QRS complex in 2022 (Fig. 1A1–D1 and A2–D2). Follow-up clinical course, ECG, and TTE parameters are summarized in Table 1.

**Table 1:** Description of follow-up clinical course, electrocardiogram, and transthoracic echocardiography parameters

Parameters	2018.03∗	2018.06	2020.12	2022.09
NT-proBNP, pg/mL	7652	171.3	408	/
NYHA functional class	II	I	I	II
P-wave duration, ms	132	138	163	UN
PR segment, ms	125	163	170	Retrograde P'
QRS duration, ms	91	/	100	97
LVEDV, mL	108.65	78.88	76.3	74.44
LVEDVi, mL/m^2^	78.73	57.16	55.29	53.94
LVEDD, mm	54	49	49	46
LVEDDi, mm/m^2^	39.13	35.51	35.51	33.33
LVEF, %	44	55.7	69	62
LA, mL	43.38	43	93.7	91.75
RA, mL	42.74	42.18	50.46	74.17
MR jet area/LAA, %	22.8	13.5	50.9	65.7
LA-GLS, %	21.39	20.98	15.65	5.83
LA-FAC, %	33.08	31.96	17.99	13.37
RA-GLS, %	30.05	25.08	26.25	10.31
RA-FAC, %	30.95	27.86	15.41	8.26

FAC, fractional area change; GLS, global longitudinal strain; LA, left atrium; LAA, left atrial area; LVEDD, left ventricular end-diastolic diameter; LVEDDi, LVEDD indexed to body surface area; LVEDV, left ventricular end-diastolic volume; LVEDVi, LVEDV indexed to body surface area; LVEF, left ventricular ejection fraction; MR, mitral regurgitation; NT-proBNP, N-terminal pro-B-type natriuretic peptide; NYHA, New York Heart Association; RA, right atrium.

∗ The parameters are post-permanent pacemaker implantation.

Given these findings, hemodynamic evaluation under varying pacing outputs was performed. Simultaneous ECG and TTE assessments were conducted in dual-chamber pacing with atrial sensing and tracking mode with different settings. At lower output (5V/0.4 ms, paced AV delay [pAVD] 150 ms), retrograde P' waves were observed post-QRS no atrial capture was apparent (Fig. 1E1 and E2); and TTE transmitral inflow Doppler revealed E/A waves fusion (Fig. 1E3), and moderate-to-severe systolic MR (effective regurgitant orifice area [EROA] measured by LBBB-induced cardiomyopathy proximal isovelocity surface area method at 0.42 cm^2^) combined with diastolic MR (Fig. 1E4). At higher output (7V/1.0 ms, pAVD, 350 ms), anterograde P waves were restored with atrial capture, but significant atrial pacing delays (AP-P interval, 200 ms) were noted (Fig. 1, F1 and F2). Distinct E and A waves can be detected (Fig. 1F3). Quantitative TTE analysis showed reduced MR severity (Fig. 1F4**;** EROA measured by the proximal isovelocity surface area method at 0.28 cm^2^).

As the current MR is not causing significant symptoms, and increasing atrial pacing output mitigates its severity, the patient continues with regular outpatient follow-up. Mitral transcatheter edge-to-edge repair may be considered in symptomatic patients with severe secondary MR and an LVEF > 50% (usually atrial FMR) who remain symptomatic despite optimized guideline-directed medical therapy, have high surgical risk according to the heart team, and are suitable for the intervention.^1^ Given the patient’s preserved LV dimensions, the absence of significant leaflet tethering, and the central MR jet, if MR should worsen or symptoms develop, mitral transcatheter edge-to-edge repair may be considered.

## Discussion

FMR is a complex entity requiring tailored diagnostic and therapeutic approaches. This case uniquely illustrates how distinct FMR mechanisms can manifest sequentially in a single patient.

Initially, the patient presented with ventricular FMR secondary to LV systolic dysfunction caused by LBBB-induced cardiomyopathy. LBBB typically causes dyssynchronous ventricular contraction, leading to inefficient LV systolic performance and MR due to papillary muscle dysfunction and mitral valve (MV) geometric distortion.^2^ Left bundle branch pacing effectively improved electrical resynchronization and reduced FMR severity in this case, likely by correcting subvalvular traction forces and promoting LV reverse remodelling.^3^

Despite this initial improvement, progressive P-wave changes on ECG, indicative of loss of atrial capture and evolving atrial dysfunction, were observed. Concurrently, MR worsened alongside LA enlargement and deteriorating LA function, despite preserved LV size and systolic function. This progression suggests the emergence of atrial FMR, a recently recognized secondary MR subtype characterized by preserved LV function, mitral annular dilation, LA enlargement, and loss of MV leaflet concavity.^4^

In addition, abnormal AV synchrony significantly contributed to MR exacerbation. When atrial activation occurs during or after ventricular systole, atrial booster pump function is compromised. MV closure coinciding with LA contraction elevates LA pressure. Atrial pathology leading to capture failure creates an abnormal AV activation sequence, which further stresses the atria, creating a vicious cycle of worsening atrial function.

This case is important, as it highlights the presence of diastolic MR, reflecting reversal of the left AV pressure gradient. Initial diastolic MR was likely due to dyssynchronous LV relaxation caused by LBBB-induced cardiomyopathy. Later diastolic MR was exacerbated by retrograde atrial activation and elevated LV diastolic pressure. Concomitant moderate aortic regurgitation contributed to LV volume overload and increased filling pressures, further aggravating diastolic MR.^5^

This case exemplifies the sequential evolution of distinct FMR subtypes within one patient, underscoring the necessity for comprehensive hemodynamic and imaging assessments to guide appropriate management. Further research into tailored treatment strategies and long-term outcomes is warranted.

**Novel Teaching Points**

• Multiple mechanisms can underlie FMR, and these mechanisms may evolve sequentially in a single patient.
• Recognizing the specific etiology driving FMR is crucial for accurate diagnosis and effective management of cardiovascular disease.
